# Scleritis Following Pterygium Excision: Infection, Autoimmunity, or Both?

**DOI:** 10.7759/cureus.17736

**Published:** 2021-09-05

**Authors:** Fatima Zahra Mabrouki, Rachid Sekhsoukh

**Affiliations:** 1 Ophthalmology, Mohammed VI University Hospital / Faculty of Medicine and Pharmacy, Oujda, MAR

**Keywords:** ophthalmology, scleritis, pterygium excision, infection, autoimmunity

## Abstract

The term scleritis refers to a heterogeneous group of disorders characterized by a chronic inflammatory response centered on the sclera and often involving adjacent structures such as the episclera, cornea, and uvea. It is an uncommon but severe, painful, and potentially blinding ocular disease and may occasionally result in perforation of the globe. Through a case report of severe scleritis that occurred after pterygium surgery, the authors expose diagnostic difficulties and emphasize the need for a meticulous diagnostic approach in such a condition.

## Introduction

Infectious and necrotizing scleritis are two subtypes of scleritis. Early antibiotic treatment in infectious scleritis and systemic immunosuppression in necrotizing scleritis are essential for preventing the infection from spreading in other adjacent ocular structures and preserving vision. Through a case report of severe scleritis that occurred after pterygium surgery, authors expose diagnostic difficulties and emphasize the need for a meticulous diagnostic approach in such a condition.

## Case presentation

**Figure 1:**
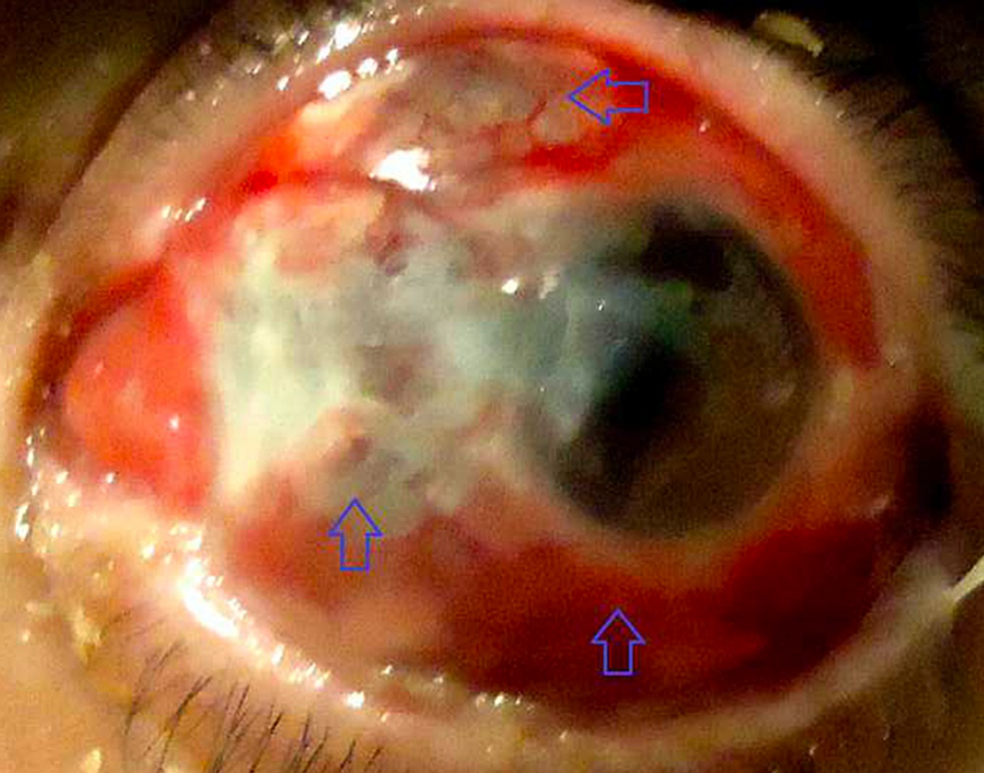
Photograph of the left eye obtained eight days after pterygium removal surgery Marked conjunctival and scleral injection, many small and large, purulent-appearing subconjunctival abscesses with sectoral subconjunctival necrosis and focal full thickness necrosis at the surgical site of pterygium excision are apparent.

A 70-year-old man was referred for the evaluation of blurred vision and a red and inflamed left eye that began seven days after uncomplicated pterygium excision. Her medical history was significant for type 2 diabetes and arterial hypertension and was negative for eye surgery and systemic autoimmune disease. The patient denied recent ocular trauma, night sweats, fevers, skin rashes, or joint pains. Examination revealed visual acuity of 7/10 in the right eye and hand motion in the left eye. Slit-lamp examination showed large necrosis at the surgical site of pterygium excision with many small and large purulent appearing subconjunctival abscesses with a conjunctival and scleral injection of the left eye (Figure 1). There was a slight Tyndall phenomenon in the anterior chamber and moderate corneal edema with a number of blood vessels in the cornea and iridocapsular synechiae. Extraocular motility examination revealed mild limitation of abduction in the left eye with normal adduction. Intraocular pressure was normal. Fundus examination was not possible because of corneal edema. The right eye was in perfect condition. A general physical examination was performed, and there was no evidence of systemic vasculitis or arthritis. Surgical debridement of all the subconjunctival abscesses was realized. A culture specimen of this tissue was obtained and was negative. Associated systemic conditions were investigated with chest X-ray and blood tests, including erythrocyte sedimentation rate (ESR), complete blood count, serum uric acid, syphilis serology, serum antibody screen (rheumatoid factor, antinuclear antibodies, anti-DNA antibodies), circulating immune complexes, all of which were normal, apart from a mildly elevated ESR at 18mm/h. At this stage, the patient was suspected as having scleritis following pterygium surgery without any sound argument for infectious or autoimmune origin. In view of the foregoing and because of the poor prognoses of both presentations, the patient was started on high-dose intravenous steroid therapy: methylprednisolone 1000 mg/d for three days in combination with Ceftriaxone 2 g/day and hourly topical administration of vancomycin-ceftazidime. There was an initial symptomatic improvement but the size of lesions did not increase. Four days later, the patient was commenced on systemic and topical antifungals (voriconazole) with a dramatic resolution of the scleral inflammation and pain in three days. Complete remission of scleral inflammation was observed over six weeks with residual areas of scleral thinning.

## Discussion

The difficulty of this report was to distinguish between infectious scleritis and noninfectious autoimmune scleritis, which is imperative since the treatment regimens for the first one, antibiotics, is contrary to the treatment for the second, which consists of systemic immunosuppressants [1].

The term scleritis represents a heterogeneous nosological entity of disorders characterized by a chronic inflammation response centered on the sclera and often involving adjacent structures such as the episclera, uvea, and cornea. It is a rare but severe ocular disease, painful and potentially blinding, and may occasionally result in perforation of the globe. The disease can be classified as infectious, autoimmune, or idiopathic scleritis. Approximately 5% to 10% of patients have infectious scleritis (e.g. syphilis, herpes simplex virus (HSV), varicella-zoster virus (VZV)), about 40% have a rheumatic disease, and the other 50% have no associated infectious or rheumatic disease [2].

Surgically induced scleritis (SIS) is the most severe and destructive form of scleritis. It has been described following various ocular surgeries like strabismus correction, cataract extraction, trabeculectomy, scleral buckle, pars plana vitrectomy, pterygium excision, and diode laser cyclophotocoagulation. It can be necrotizing and localized surgically induced necrotizing scleritis (SINS) or non-necrotizing and diffuse [3]. This entity is known to occur commonly in patients with systemic illnesses such as diabetes mellitus, thyroid disorders, Wegener’s granulomatosis, relapsing polychondritis, rheumatoid arthritis, systemic lupus erythematosus, polyarteritis nodosa, and inflammatory bowel disease, and as such, these patients should benefit from systemic evaluation in order to help identify an autoimmune disorder [4-5]. In our patient, we failed to identify a systemic disease. SINS occurring after pterygium surgery is a rare but relatively well-known entity that can occur from the first postoperative day to 40 years postoperatively. It has been frequently reported following the use of adjuvant mitomycin/ thiotepa or beta irradiation or bare sclera technique. indeed, the use of antiproliferative agents or irradiation can be the cause of obliterative endarteritis or inhibition of endothelial proliferation resulting in local ischemia and necrosis [5-7]. In the absence of the above, scleral necrosis was attached to the excessive use of cautery particularly in the bare sclera technique [7]. In our case, no radiation or mitomycin C was used, and cauterization was kept to a minimum during surgery. Treatment of SINS after pterygium surgery has included immunosuppression with systemic steroids, cyclophosphamide, or tacrolimus, and surgical intervention, including resection of necrotic tissue, amniotic membrane transplantation, and scleral or corneal tissue patch grafts [8]. The exact pathogenesis of SINS is unknown, although available evidence suggests that this is an immune-mediated process, which is responsive to systemic corticosteroids and/or immunosuppressive agents [7].

Infectious scleritis following pterygium surgery is also a well-documented association. Pseudomonas accounts for the majority of infections although a variety of bacteria, fungi, and atypical organisms have been described [1]. The relationship between SINS and infection remains obscure, despite an abundance of reports describing both infectious and autoimmune postoperative scleral necrosis. Many consider SINS to be a diagnosis of exclusion, not applicable to cases of infectious necrosis. Some suggest that SINS can be caused by infection, whereas others believe it may predispose to secondary infection [1]. In our patient, different diagnostic scenarios were considered. Although negative cultures, postoperative infectious scleritis was the first plausible diagnosis seeing the early onset of inflammation following surgery (7 days after surgery) and the initial appearance of the conjunctival abscesses (Figure 1). However, initially, the patient did not respond effectively to antimicrobial therapy. On the other hand, the lack of response to systemic and topical antibiotics, the rapidly progressive nature of scleritis with the invasion of the adjacent normal sclera with nodule formation, all suggested that inflammation is the main mechanism, not an infection. Furthermore, in view of the good resolution of inflammation under systemic and topical antifungals drugs, the fungal origin was also discussed, and hence, initial systemic antibiotic therapy failed to control what was suspected primarily to be a microbial or autoimmune process. Finally, it is possible that the initial infection may have triggered the release of inflammatory mediators, which could lead to the non-resolution of inflammation quickly, but it is very rare that non-infectious autoimmune scleritis is so severe and rapidly progressing to include adjacent areas of the normal sclera.

## Conclusions

Our case maintains discussion and debate on the mystery of scleritis pathogenesis after eye surgery and highlights the need for a meticulous approach in a still unresolved entity because early diagnosis and prompt treatment are imperative to reduce ocular morbidity and prevent vision loss.
